# Neurocognitive outcomes in adolescents with and without four weeks of cannabis abstinence: a randomized clinical trial using contingency management

**DOI:** 10.3389/fpsyt.2025.1723633

**Published:** 2025-12-26

**Authors:** Randi M. Schuster, Meghan A. Costello, Kevin Potter, Matteo Torquati, Jodi M. Gilman, A. Eden Evins

**Affiliations:** 1 Department of Psychiatry, Massachusetts General Hospital, Boston, MA, United States; 2 Harvard Medical School, Boston, MA, United States; 3 University of Miami Miller School of Medicine, Miami, FL, United States

**Keywords:** adolescence, cannabis, executive function, cognitive recovery, abstinence intervention, contingency management

## Abstract

**Background:**

As adolescent cannabis use becomes more common, questions remain about the potential for neurocognitive recovery after stopping cannabis use.

**Method:**

This study examined whether short-term abstinence from cannabis leads to cognitive improvements. A total of 238 adolescents (ages 13–19; 51% female; 55% White, 18% Black, 9% Asian, 18% other races) from Greater Boston participated, including 154 adolescents who regularly used cannabis (CB) and 84 adolescents with no cannabis use (NU). Participants who used cannabis were randomized to incentivized abstinence (CB-Abst) or a non-contingent monitoring control (CB-Mon). Non-users completed four weeks of monitoring (NU). Participants completed tests of executive function, memory, and attention weekly for four weeks.

**Results:**

At baseline, CB adolescents demonstrated worse verbal memory and processing speed than NU on (p <.006). CB-Abst performance was similar to that of the NU group at week 4. At week 4, those in the CB-Abst group showed greater improvements in inhibitory control compared to the CB-Mon group (β = -10.9, p = .037). There were no significant differences between CB groups in memory or attention task performance at week 4. Exploratory analyses revealed modest gains across all groups in some tasks.

**Discussion:**

Brief cannabis abstinence may be associated with improvements in executive function among adolescents, supporting the idea of neurocognitive recovery, which has important implications for treatment, prevention, and public health policies.

## Introduction

Cannabis is among the most widely used substances among adolescents, with nearly half reporting lifetime use by age 18 (1, 2). Regular use is common, with 12% of 12th graders in 2023 reporting daily cannabis use for at least one month (3). Growing commercialization has increased youth access to cannabis products (4, 5) and may lead to perceptions that cannabis is safe (6). Although adolescent cannabis use, especially early and frequent use, is linked to adverse outcomes (7), the impact of adolescent cannabis exposure on cognitive functioning remains unclear. As adolescence is a key period of brain development (8–11), understanding how cannabis affects neurocognitive functions, through controlled studies assessing the effect of ongoing cannabis exposure and abstinence in youth, is critically important (1).

Adolescent cannabis use has been linked to difficulties in verbal working memory (12–14), processing speed (15, 16), and executive functioning (12, 17–21). Much of this evidence comes from cross-sectional studies that generally demonstrate poorer neurocognitive performance amongst adolescents who use cannabis (CB) than their non-using peers (NU). Longitudinal studies suggest that early and frequent cannabis exposure predicts declines in cognitive functioning over time (22). Controlled trials are needed to assess whether the effect of cannabis is causal for cognitive deficits and rule in or out the *common liability hypothesis*, that preexisting vulnerabilities such as genetic risk, environmental adversity, or early cognitive weaknesses may contribute to both cannabis use and poorer neurocognitive performance.

Abstinence studies provide an important experimental approach to assess direct effects of cannabis exposure on cognition. Evidence from adults indicates the brain can recover: a meta-analysis of 45 studies found neural and functional improvements after stopping substance use, including improvement in executive functions (i.e., cognitive flexibility and decision-making as assessed by the Wisconsin Card Sorting and Iowa Gambling Tasks; 23). Whether similar recovery occurs in adolescents is less clear. Adolescence is a developmental stage characterized by both increased neuroplasticity, which may help repair damage from harmful exposures (24), and greater vulnerability due to rapid neural changes (25). Early abstinence studies in youth offer cautious optimism. Non-randomized trials show improvements in sustained attention after two weeks of abstinence (26) and in memory after three weeks (27). In a trial of N-acetylcysteine, teens who achieved four to eight weeks of abstinence showed improvements in verbal memory and psychomotor speed (28). Our group reported improved verbal encoding after one week of cannabis abstinence in young adults (29). These findings highlight the potential of randomized adolescent abstinence trials to clarify the potential for neurocognitive recovery after stopping cannabis use in youth.

### The current study

This longitudinal trial enrolled adolescents who used cannabis at least weekly (CB) and a comparison group of adolescents who reported no regular or current cannabis use (NU). CB adolescents were randomly assigned to four weeks of incentivized cannabis abstinence (CB-Abst) or monitoring with usual cannabis use (CB-Mon). We conducted weekly cognitive assessments over four weeks to compare the magnitude and timing of cognitive changes between adolescents assigned to incentivized cannabis abstinence and those monitored without an abstinence requirement. We hypothesized that CB-Abst would demonstrate greater improvements in memory, executive functioning, and attention than CB-Mon.

## Methods

### Participants

Study [NCT03276221] enrollment took place between August 2017 and September 2022. Participants (N = 238) were adolescents aged 13 to 19, recruited through community advertisements, direct outreach to middle and high schools in Greater Boston, and peer referrals. All participants spoke English, were medically healthy, endorsed no neurodevelopmental delays (including, but not limited to, Autism Spectrum Disorder, Intellectual Disability, and Down Syndrome), and were considered able to safely participate. CB adolescents (N = 154) reported at least weekly cannabis use, used cannabis within one week before screening, were willing to be randomized to 30 days of cannabis abstinence, and were not seeking assistance to or planning to stop regular cannabis use. NU adolescents (N = 84) reported no more than five cannabis use occasions lifetime and none in the prior year or before age 16.

### Procedures

Procedures were approved by the Mass General Brigham Institutional Review Board. Written informed consent was obtained from participants aged 18 years or older, while those under age 18 provided written parental consent and participant assent. Participants then completed a baseline assessment over two visits. Participants reporting regular cannabis use were then randomly assigned to 4 weeks of incentivized abstinence using contingency management (CB-Abst; n=125) or 4 weeks of monitoring without incentives for abstinence (CB-Mon; n=94). Randomization was stratified by sex (male vs. female), age (13–16 years vs. ≥ 17 years), and average frequency of cannabis use (1 day per week vs. ≥ 1 day per week). All participants completed study visits, occurring approximately on study days 3, 5, 7, 14, 21, and 28 post-baseline.

### Contingency management protocol

This study used a voucher-based contingency management (CM) procedure (29–32) for participants randomized to CB-Abst that offered escalating payments as incentives for biochemically verified cannabis abstinence over a 4-week period. At the baseline visit, all CB participants completed an abstinence contract with a study staff member, which outlined expected behavior changes over the 4-week period, the payment schedule, and contingencies if they were randomized to CB-Abst (32). At the end of the baseline visit, participants assigned to CB-Abst were asked to immediately stop cannabis use for four weeks. CB-Mon participants were not required to change their substance use habits. CB-Abst participants who resumed cannabis use within the first week of abstinence were given one opportunity to restart the abstinence protocol and remain in the study. Those who resumed cannabis after the first week of abstinence were excluded.

During the intervention phase, CB-Abst participants received incentives through a two-part system that included fixed payments for study visit attendance and increasing payments for biochemically verified ongoing cannabis abstinence. After completing the 4-week abstinence period, CB-Abst participants were no longer required to abstain from cannabis and were paid for attending a post-baseline follow-up visit (32). CB-Mon and NU participants were paid on an escalating schedule solely for study visit attendance. The payment schedule is shown in **Table 1**. Participants were paid via reloadable debit cards through Clinical Trials Payer (CT Payer), a secure web-based platform that ensures HIPAA and HITECH compliance for clinical trial payments. Payments were made on the day of the visit for attendance and after receipt of quantitative urinalysis results confirming abstinence, which typically occurred within 2–5 days after the study visit. Among participants assigned to the CB-Abst group, 6 resumed cannabis use. *Post-hoc* t-test analyses identified a trend toward higher CUDIT scores among CB-Abst participants who did not maintain abstinence compared to those who did (mean difference in scores = 4.5; p = 0.05), suggesting that higher cannabis dependence posed challenges in maintaining abstinence in the context of the contingency management protocol.

**Table 1:** Remuneration schedule for participants.

Visit		Contingency management (cannabis users; CB-Abst)		Monitoring (cannabis users; CB-Mon)		Non-users (NU)
		Attendance	Abstinence	Attendance	Abstinence	Attendance
1	Baseline part I	$10	–	$10	–	$10
2	Baseline part II	$10	–	$15	N/A	$15
3	3 days	$10	$15	$20	N/A	$20
4	4 days	$10	$30	$25	N/A	$25
5	1 week	$10	$45	$30	N/A	$30
6	2 weeks	$10	$60	$35	N/A	$35
7	3 weeks	$10	$75	$40	N/A	$40
8	4 weeks	$10	$90	$45	N/A	$45
9	4-week follow-up	$10 +$15*	–	$50	N/A	50
Max subtotal		$105	$315	$270	N/A	$270
Max total		$420		$270		$270

*An additional $15 for completing study procedures.

### Bio-verification of cannabis abstinence

Participants provided a urine sample at each study visit for 11-nor-9-carboxy-tetrahydrocannabinol (THCCOOH) concentration determination via liquid chromatography-tandem mass spectrometry, with a quantitation limit of 5 ng/mL and an upper linearity limit of 500 ng/mL (Dominion Diagnostics, Richmond VA). When necessary, samples were subjected to serial dilutions to ensure values fell within the assay’s linear range (5 ng/mL–500 ng/mL). THCCOOH concentrations were adjusted based on creatinine levels (measured in mg/dL), generating a THCCOOH to creatinine ratio for each time point (CN-THCCOOH; measured in ng/mg). To verify self-reported abstinence, CN-THCCOOH ratios for specimen pairs collected ≥48 hours apart were compared to an expected CN-THCCOOH ratio determined using a statistical model developed by Schwilke and colleagues (33). A specificity threshold of 95% was adopted, allowing for a false positive rate of 2.5%. Relevant to this algorithm, the sample reported days since last use prior to initiating abstinence. CB-Abst participants reported, on average, 1.4 days since last cannabis use when starting abstinence (*SD* = 1.5 days, median=1 day, range=0–7 days).

### Cognitive functioning assessment

#### Cognitive functioning: executive functioning, memory, and attention

The Cambridge Neuropsychological Test Automated Battery (CANTAB) was used to assess cognition at baseline and weekly for four weeks. Eight modules evaluated executive function (Multitasking Test, One-Touch Stockings of Cambridge, Stop Signal Task, Spatial Working Memory), memory (Paired Associates Learning, Spatial Span, and Verbal Recognition Memory), and attention (Rapid Visual Information Processing; see **Supplementary Table 1**. for descriptions of all CANTAB modules used;).

### Self-report measures

#### Demographic and background information

Baseline assessments included participant-reported age, sex, gender, and racial identity, Cannabis Use Disorder Identification Test–Revised (CUDIT-R: 34), Alcohol Use Disorders Identification Test (AUDIT; 35), 90-day Timeline Follow-back interview to approximate the quantity and frequency of cannabis and alcohol use, Adolescent Psychotic-Like Symptom Screener (APSS; 36), Screener for Child Anxiety Related Disorders (SCARED; 37), and the Mood and Anxiety Symptoms Questionnaire (MASQ; 38).

### Analytic plan

The initial implementation of analyses was analyst-blind (39). We examined all outcomes using regression models fitted with generalized estimating equations (GEE), a reliable alternative to maximum likelihood estimation for mixed effects models (40). We assumed data were clustered by subjects and that the observations within each study visit for a subject were uncorrelated (the GEE method is robust to misspecification of the correlation structure among a subject’s observations). Continuous outcomes, such as latencies and reaction times, were analyzed using linear regression. Bounded count outcomes, such as the number of errors or correct responses, were analyzed with binomial regression to address potential non-normality issues. Outcomes were examined across post-baseline study weeks 1 to 4. The primary confirmatory effect of interest for each outcome was a dummy-coded contrast between CB-Mon (the reference group, coded as 0) and CB-Abst (coded as 1), testing whether a constant effect of abstinence exists across all time points. A sensitivity analysis evaluated whether a group-by-time interaction term was necessary. Models included covariates for linear trends and baseline outcome score. Additional sensitivity analyses incorporated covariates for age (years), sex (male vs. female), and baseline CUDIT-R scores. P-values were adjusted for multiple comparisons using the Bonferroni method within each module. Clinical significance was assessed by computing standardized effect sizes, estimated by dividing the effect estimate (on the original measurement scale) by the pooled standard deviation of the outcome during the baseline visit.

Rates of missingness on all primary variables are presented in **Supplementary Table 2** (range = 8.2-11.6%). Missing outcome data were imputed 48 times using chained equations (MICE; 41; version 3.14.0; 42).) with linear regression and predictive mean matching. Results were then pooled following Rubin’s rule. Participants were included in the analysis if they had data from at least one post-baseline visit and biochemically confirmed abstinence. Covariates for imputing missing values included baseline and non-missing post-baseline outcome scores, age in years, biological sex at birth, baseline CUDIT-R scores, and years of cannabis exposure. A sensitivity analysis was conducted including only participants with complete data. Analyses were performed using R (version 4.1.1; 43) and RStudio (version 2020.9.0.351; 44). Data were prepared with R packages ‘dplyr’ (version 1.0.7; 45) and ‘tidyr’ (version 1.1.4; 46). Models were fitted with the R package ‘geepack’ (version 1.3.4; 47). Reproducible code and de-identified data were organized using the R package ‘targets’ (version 0.8.1; 48).

## Results

### Sample descriptives

Of 238 participants screened, 201 participants were eligible and completed baseline measures, 132 (65.7%) used cannabis at least weekly (CB) (M = 4.2, SD = 2.2 days per week) and 69 (34.3%) did not use cannabis (NU). Groups differed significantly on age and alcohol use and no other baseline characteristics, (see **Table 2**, **Supplementary Table 1**).

**Table 2:** Descriptive statistics and group comparisons at baseline.

Domain	Measure	CB-Abst	CB-Mon	NU	Significant group differences
	Sample size	54	66	69	
	Completed intervention phase; % (n)	83.3 (45)	89.4 (59)	92.8 (64)	
	Age; M (SD)	18.3 (1.4)	18.1 (1.5)	17 (1.8)	CB-Mon > NU; CB-Abst > NU
	Biological sex; % (n)				
	- Female	48.1 (26)	47 (31)	58 (40)	
	- Male	51.9 (28)	53 (35)	42 (29)	
	Race; % (n)				
	- American Indian/Alaska Native	1.9 (1)	1.5 (1)	0 (0)	
	- Asian	7.4 (4)	10.6 (7)	8.7 (6)	
	- Black/African American	18.5 (10)	15.2 (10)	20.3 (14)	
	- More than one race	11.1 (6)	16.7 (11)	5.8 (4)	
	- White	57.4 (31)	51.5 (34)	56.5 (39)	
	- Not listed	3.7 (2)	4.5 (3)	8.7 (6)	
	Hispanic/Latino(a); % (n)	9.3 (5)	24.2 (16)	13 (9)	
	Mental health symptoms				
	- APSS; M (SD)	0.1 (0.6)	0.3 (0.8)	0.1 (0.5)	
	- MASQ; M (SD)	121.6 (26.4)	122.1 (28.4)	112.4 (28.3)	
	- SCARED GAD; M (SD)	6 (4.4)	5.9 (4.3)	6.4 (4.6)	
	Substance use				
	- Years w/cannabis use; M (SD)	3.3 (1.3)	3.4 (1.5)		
	- Days/wk cannabis use; M (SD)	3.6 (2.8)	3.3 (2.6)		
	- THCCOOH levels; M (SD)	211.8 (338)	389.6 (745)		
	- CUDIT-R; M (SD)	13.7 (5.4)	14.1 (5.5)		
	- Days/wk alcohol use; M (SD)	0.7 (0.8)	0.4 (0.8)	0.1 (0.2)	CB-Abst > CB-Mon; CB-Mon > NU; CB-Abst > NU
	- AUDIT; M (SD)	5.4 (4.8)	5.9 (5)	1.9 (2.5)	CB-Mon > NU; CB-Abst > NU
Memory (PAL)	Total number of adjusted errors	6.4 (6.3)	5.9 (5.4)	7.1 (6.0)	
	First attempt memory score	15.7 (2.8)	15.6 (3.1)	15.1 (3.4)	
Memory (SSP)	Forward span length	7.0 (1.5)	7.3 (1.4)	7.1 (1.3)	
	Reverse span length	6.8 (1.6)	6.7 (1.4)	6.7 (1.4)	
Memory (VRM)	Immediate recall - total correct	6.0 (2.4)	6.7 (1.9)	7.4 (2.2)	CB-Mon < NU; CB-Abst < NU
	Delayed recall - total correct	6.5 (2.8)	6.9 (2.5)	7.6 (2.3)	CB-Mon < NU; CB-Abst < NU
	Immediate recognition - total correct	32.4 (3.8)	33.2 (2.0)	32.4 (2.6)	
	Delayed recognition - total correct	32.4 (2.7)	32.7 (2.5)	32.2 (2.8)	
Executive Functioning (MTT)	Total incorrect	5.0 (5.5)	6.0 (8.3)	5.8 (5.9)	
	Median response latency (ms)	557 (87)	548 (90)	586 (85)	CB-Mon < NU; CB-Abst < NU
	Median incongruency cost (ms)	40 (33)	50 (32)	55 (46)	
	Median multitasking cost (ms)	159 (107)	134 (75)	163 (100)	
Executive Functioning (OTS)	Problems solved on first choice	11.9 (1.9)	11.2 (2.2)	11.3 (2.6)	
	Median latency to first correct choice (ms)	9714 (3032)	9161 (3370)	9682 (3454)	
Executive Functioning (SST)	Stop signal reaction time (ms)	224 (47)	220 (45)	232 (62)	
Executive Functioning (SWM)	Total number of between errors	5.1 (6.2)	6.0 (7.2)	8.6 (8.3)	CB-Mon < NU; CB-Abst < NU
	Strategy for 6–8 box conditions	6.4 (2.6)	6.1 (2.8)	6.9 (2.3)	CB-Abst > CB-Mon; CB-Mon < NU; CB-Abst < NU
Attention (RVP)	Discriminability - A’	0.92 (0.05)	0.91 (0.05)	0.89 (0.07)	CB-Mon > NU; CB-Abst > NU
	Median response time for hits (ms)	444 (55)	434 (63)	440 (54)	

APSS, Adolescent Psychotic-Like Symptom Screener; MASQ, Mood and Anxiety Symptoms Questionnaire, SCARED GAD, Screener for Child Anxiety Related Disorders – Generalized Anxiety Disorder; CUDIT, Cannabis Use Disorder Identification Test; AUDIT, Alcohol Use Disorder Identification Test; PAL, Paired Associates Learning; SSP, Spatial Span; VRM, Verbal Recognition Memory; MTT, Multitasking Test; OTS, One-Touch Stockings of Cambridge; SST, Stop Signal Task; SWM, Spatial Working Memory; RVP, Rapid Visual Information Processing.

**Figure 1:**
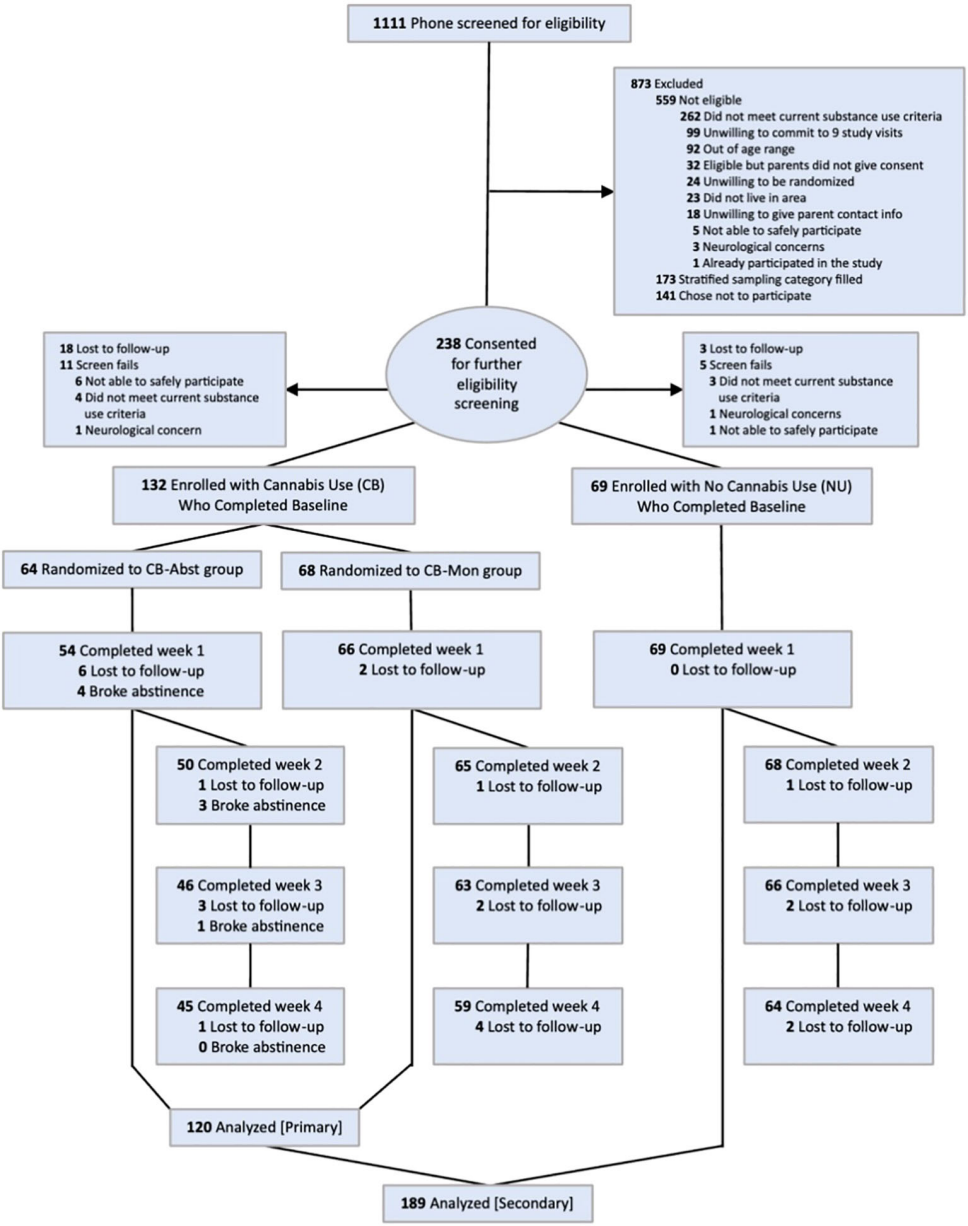
Study consort diagram.

Among CB, 64 (48.5%) were randomized to CB-Abst and 68 (51.5%) to CB-Mon; 120 CB participants completed at least one post-baseline study visit and were included in the analyses (CB-Abst: 54, 84.4% of those randomized; CB-Mon: 66, 97.1% of those randomized). Among CB-Abst participants included in the analysis, 48 (88.9%) maintained biochemically-verified cannabis abstinence throughout the intervention period. Among NU 69 (100%) completed at least one post-baseline study visit and were included in analyses. See Consort Diagram, **Figure 1**.

### Baseline cognition

First, we note comparisons between baseline CB and NU groups because these groups were not randomly assigned. Comparing baseline NU and CB groups, NU had higher total correct for immediate recall (p = 0.001) and delayed recall (p = 0.006) on the VRM and faster median response times on the MTT (p = 0.012) and more errors (p = 0.004), yet better strategy on SWM (p = 0.007).

Second, we note comparisons between CB-Abst and CB-Mon, to characterize these randomly assigned groups. CB-Abst and CB-Mon were comparable across all baseline measures of memory (p’s > 0.16), attention (p’s > 0.35), and executive functioning (p’s > 0.06; see **Table 2**, **Supplementary Table 1**), except that CB-Abst had a better strategy on the SWM (p = 0.048).

### Effects of abstinence

**Figure 2:**
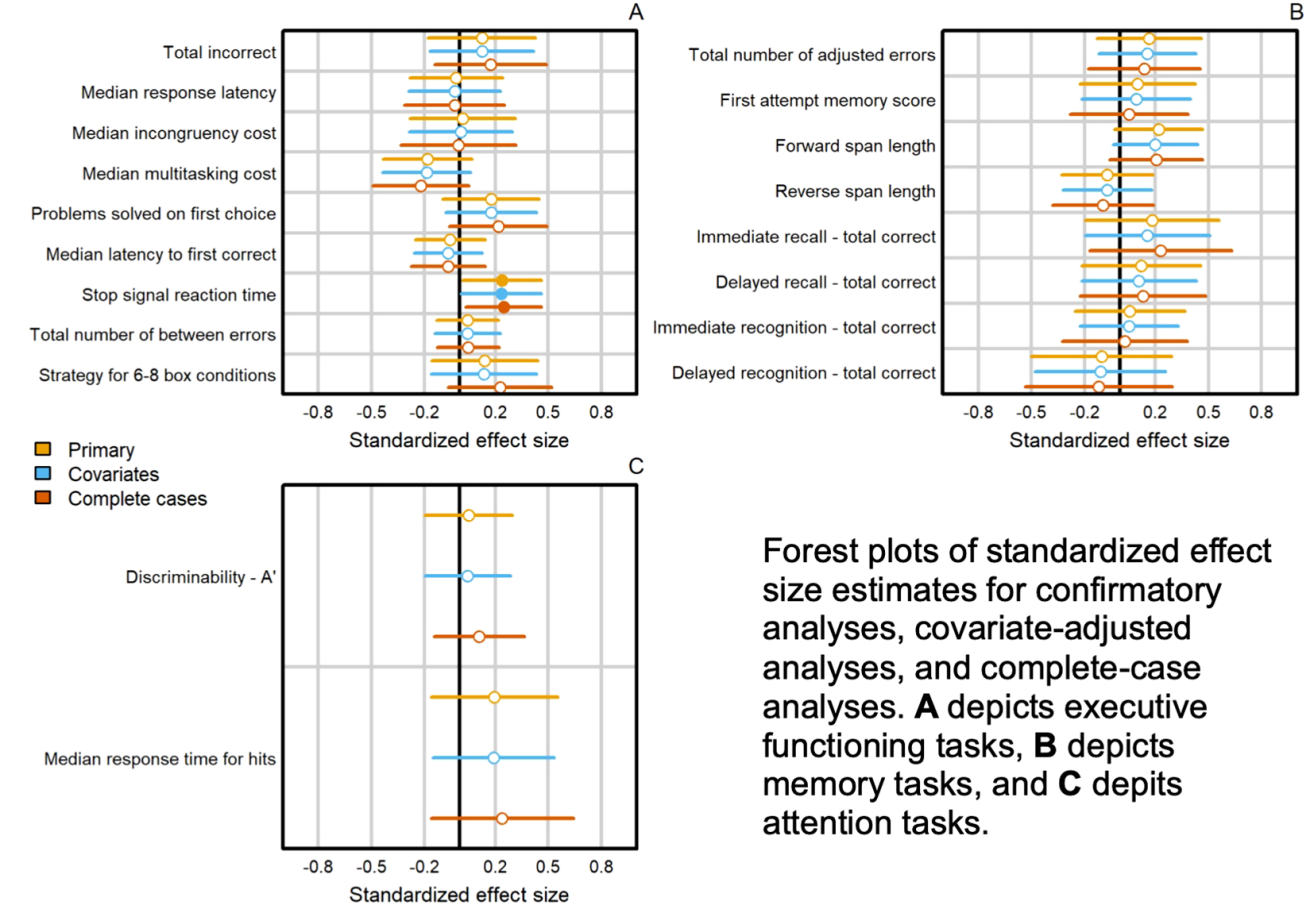
Summary of confirmatory analyses: effects of abstinence on neurocognitive functioning among adolescents with cannabis use.

Our confirmatory contrast of interest was the average post-baseline difference between CB-Abst and CB-Mon. CB-Abst had significantly faster average reaction times on the SST module over the 4 post-baseline visits compared to CB-Mon (β = -10.9 [SE = 5.2], p = 0.037; ES = -0.24 [-0.46 to -0.01]; **Table 3**; **Figure 2**). Analyses adjusted for age, sex, and baseline CUDIT-R scores (p = 0.045) and sensitivity analysis with complete cases (p = 0.02) confirmed the finding of faster reaction times on the SST among CB-Abst compared to CB-Mon. There were no other group differences in the remaining measures of executive functioning, memory, or attention (all p > 0.05).

**Table 3:** Confirmatory and sensitivity analysis results.

Analysis	Module	Outcome	Test
Executive Functioning			
MTT	Total incorrect	Primary	β = -0.12 [SE = 0.12]; p = 0.312 (α = 0.0125); ES = -0.12 [-0.43 to 0.18]
MTT		Sensitivity: Covariates	β = -0.13 [SE = 0.12]; p = 0.295 (α = 0.0125); ES = -0.12 [-0.42 to 0.17]
MTT		Sensitivity: Complete cases	β = -0.18 [SE = 0.13]; p = 0.177 (α = 0.0125); ES = -0.17 [-0.49 to 0.14]
MTT	Median response latency (ms)	Primary	β = 1.9 [SE = 9.3]; p = 0.836 (α = 0.0125); ES = 0.02 [-0.24 to 0.28]
MTT		Sensitivity: Covariates	β = 2.7 [SE = 9.1]; p = 0.769 (α = 0.0125); ES = 0.03 [-0.23 to 0.29]
MTT		Sensitivity: Complete cases	β = 2.7 [SE = 10]; p = 0.789 (α = 0.0125); ES = 0.03 [-0.25 to 0.31]
MTT	Median incongruency cost (ms)	Primary	β = -0.5 [SE = 3.9]; p = 0.898 (α = 0.0125); ES = -0.02 [-0.31 to 0.28]
MTT		Sensitivity: Covariates	β = -0.2 [SE = 3.8]; p = 0.966 (α = 0.0125); ES = -0.01 [-0.3 to 0.29]
MTT		Sensitivity: Complete cases	β = 0.3 [SE = 4.2]; p = 0.949 (α = 0.0125); ES = 0.01 [-0.32 to 0.34]
MTT	Median multitasking cost (ms)	Primary	β = 16.5 [SE = 9.1]; p = 0.068 (α = 0.0125); ES = 0.18 [-0.07 to 0.43]
MTT		Sensitivity: Covariates	β = 17.1 [SE = 9]; p = 0.059 (α = 0.0125); ES = 0.19 [-0.06 to 0.44]
MTT		Sensitivity: Complete cases	β = 19.6 [SE = 9.6]; p = 0.042 (α = 0.0125); ES = 0.22 [-0.05 to 0.49]
OTS	Problems solved on first choice	Primary	β = 0.15 [SE = 0.1]; p = 0.150 (α = 0.025); ES = 0.18 [-0.1 to 0.45]
OTS		Sensitivity: Covariates	β = 0.15 [SE = 0.1]; p = 0.121 (α = 0.025); ES = 0.18 [-0.08 to 0.44]
OTS		Sensitivity: Complete cases	β = 0.19 [SE = 0.11]; p = 0.081 (α = 0.025); ES = 0.22 [-0.06 to 0.49]
OTS	Median latency to first correct choice (ms)	Primary	β = 179.1 [SE = 281.8]; p = 0.525 (α = 0.025); ES = 0.06 [-0.14 to 0.25]
OTS		Sensitivity: Covariates	β = 212.9 [SE = 273.5]; p = 0.436 (α = 0.025); ES = 0.07 [-0.12 to 0.26]
OTS		Sensitivity: Complete cases	β = 212.1 [SE = 295.1]; p = 0.472 (α = 0.025); ES = 0.07 [-0.14 to 0.28]
SST	Stop signal reaction time (ms)	Primary	β = -10.9 [SE = 5.2]; p = 0.037 (α = 0.05); ES = -0.24 [-0.46 to -0.01]
SST		Sensitivity: Covariates	β = -10.7 [SE = 5.3]; p = 0.045 (α = 0.05); ES = -0.23 [-0.46 to -0.01]
SST		Sensitivity: Complete cases	β = -10.7 [SE = 4.8]; p = 0.024 (α = 0.05); ES = -0.25 [-0.46 to -0.03]
SWM	Total number of between errors	Primary	β = -0.1 [SE = 0.17]; p = 0.576 (α = 0.025); ES = -0.04 [-0.22 to 0.13]
SWM		Sensitivity: Covariates	β = -0.1 [SE = 0.19]; p = 0.603 (α = 0.025); ES = -0.04 [-0.23 to 0.14]
SWM		Sensitivity: Complete cases	β = -0.12 [SE = 0.19]; p = 0.538 (α = 0.025); ES = -0.05 [-0.22 to 0.13]
SWM	Strategy for 6–8 box conditions	Primary	β = -0.15 [SE = 0.15]; p = 0.296 (α = 0.025); ES = -0.14 [-0.44 to 0.16]
SWM		Sensitivity: Covariates	β = -0.15 [SE = 0.14]; p = 0.307 (α = 0.025); ES = -0.14 [-0.43 to 0.16]
SWM		Sensitivity: Complete cases	β = -0.26 [SE = 0.15]; p = 0.088 (α = 0.025); ES = -0.23 [-0.52 to 0.07]
Memory			
PAL	Total number of adjusted errors	Primary	β = -0.19 [SE = 0.15]; p = 0.215 (α = 0.025); ES = -0.16 [-0.46 to 0.13]
PAL		Sensitivity: Covariates	β = -0.18 [SE = 0.14]; p = 0.206 (α = 0.025); ES = -0.16 [-0.43 to 0.12]
PAL		Sensitivity: Complete cases	β = -0.16 [SE = 0.16]; p = 0.335 (α = 0.025); ES = -0.14 [-0.45 to 0.18]
PAL	First attempt memory score	Primary	β = 0.1 [SE = 0.14]; p = 0.495 (α = 0.025); ES = 0.1 [-0.23 to 0.43]
PAL		Sensitivity: Covariates	β = 0.09 [SE = 0.13]; p = 0.507 (α = 0.025); ES = 0.09 [-0.22 to 0.4]
PAL		Sensitivity: Complete cases	β = 0.05 [SE = 0.14]; p = 0.725 (α = 0.025); ES = 0.05 [-0.28 to 0.38]
SSP	Forward span length	Primary	β = 0.28 [SE = 0.14]; p = 0.054 (α = 0.025); ES = 0.22 [-0.03 to 0.47]
SSP		Sensitivity: Covariates	β = 0.25 [SE = 0.14]; p = 0.062 (α = 0.025); ES = 0.2 [-0.04 to 0.44]
SSP		Sensitivity: Complete cases	β = 0.25 [SE = 0.15]; p = 0.086 (α = 0.025); ES = 0.2 [-0.06 to 0.47]
SSP	Reverse span length	Primary	β = -0.07 [SE = 0.12]; p = 0.532 (α = 0.025); ES = -0.07 [-0.33 to 0.19]
SSP		Sensitivity: Covariates	β = -0.07 [SE = 0.12]; p = 0.523 (α = 0.025); ES = -0.07 [-0.32 to 0.18]
SSP		Sensitivity: Complete cases	β = -0.1 [SE = 0.13]; p = 0.451 (α = 0.025); ES = -0.1 [-0.38 to 0.19]
VRM	Immediate recall - total correct	Primary	β = 0.09 [SE = 0.07]; p = 0.226 (α = 0.0125); ES = 0.18 [-0.19 to 0.56]
VRM		Sensitivity: Covariates	β = 0.08 [SE = 0.07]; p = 0.269 (α = 0.0125); ES = 0.16 [-0.2 to 0.51]
VRM		Sensitivity: Complete cases	β = 0.11 [SE = 0.08]; p = 0.152 (α = 0.0125); ES = 0.23 [-0.17 to 0.63]
VRM	Delayed recall - total correct	Primary	β = 0.08 [SE = 0.09]; p = 0.369 (α = 0.0125); ES = 0.12 [-0.21 to 0.46]
VRM		Sensitivity: Covariates	β = 0.07 [SE = 0.08]; p = 0.408 (α = 0.0125); ES = 0.11 [-0.22 to 0.43]
VRM		Sensitivity: Complete cases	β = 0.08 [SE = 0.09]; p = 0.363 (α = 0.0125); ES = 0.13 [-0.23 to 0.48]
VRM	Immediate recognition - total correct	Primary	β = 0.05 [SE = 0.11]; p = 0.647 (α = 0.0125); ES = 0.06 [-0.25 to 0.37]
VRM		Sensitivity: Covariates	β = 0.05 [SE = 0.1]; p = 0.635 (α = 0.0125); ES = 0.05 [-0.23 to 0.33]
VRM		Sensitivity: Complete cases	β = 0.02 [SE = 0.12]; p = 0.839 (α = 0.0125); ES = 0.03 [-0.32 to 0.38]
VRM	Delayed recognition - total correct	Primary	β = -0.06 [SE = 0.09]; p = 0.511 (α = 0.0125); ES = -0.1 [-0.5 to 0.29]
VRM		Sensitivity: Covariates	β = -0.07 [SE = 0.09]; p = 0.451 (α = 0.0125); ES = -0.11 [-0.48 to 0.26]
VRM		Sensitivity: Complete cases	β = -0.07 [SE = 0.1]; p = 0.472 (α = 0.0125); ES = -0.12 [-0.54 to 0.3]
Attention			
RVP	Discriminability - A’	Primary	β = 0.003 [SE = 0.006]; p = 0.650 (α = 0.025); ES = 0.05 [-0.2 to 0.3]
RVP		Sensitivity: Covariates	β = 0.002 [SE = 0.006]; p = 0.684 (α = 0.025); ES = 0.04 [-0.2 to 0.29]
RVP		Sensitivity: Complete cases	β = 0.006 [SE = 0.006]; p = 0.337 (α = 0.025); ES = 0.11 [-0.15 to 0.37]
RVP	Median response time for hits (ms)	Primary	β = -11.6 [SE = 9.5]; p = 0.223 (α = 0.025); ES = -0.19 [-0.55 to 0.16]
RVP		Sensitivity: Covariates	β = -11.4 [SE = 9.1]; p = 0.211 (α = 0.025); ES = -0.19 [-0.53 to 0.15]
RVP		Sensitivity: Complete cases	β = -14.2 [SE = 10.6]; p = 0.182 (α = 0.025); ES = -0.24 [-0.64 to 0.16]

Highlighted rows indicate statistically significant outcomes. Adjusted alpha using Bonferroni correction shown to right of p-values.

Sensitivity analyses checked for main effects of time and group by time interactions for CB-Abst compared to CB-Mon. For memory, by week 4, participants on average had improved forward (p = 0.01) and reverse (p = 0.01) span for the SSP module but had worse immediate (p = 0.009) and delayed (p < 0.001) recognition for the VRM module. For executive functioning, participants by week 4 had on average worse error rates (p < 0.001) but improved response times (p < 0.001), incongruency costs (p < 0.001), and multitasking costs (p < 0.001) for the MTT module, and improved accuracy (p < 0.001) and response times (p < 0.001) for the OTS module. In the attention domain, by week 4, participants on average had improved discriminability (p = 0.003) for the RVP task. Across all cognitive domains, models with a group x time interaction term did not significantly outperform a simpler model with main effects only (p’s > 0.15; see **Supplementary Table 3**).

**Figure 3:**
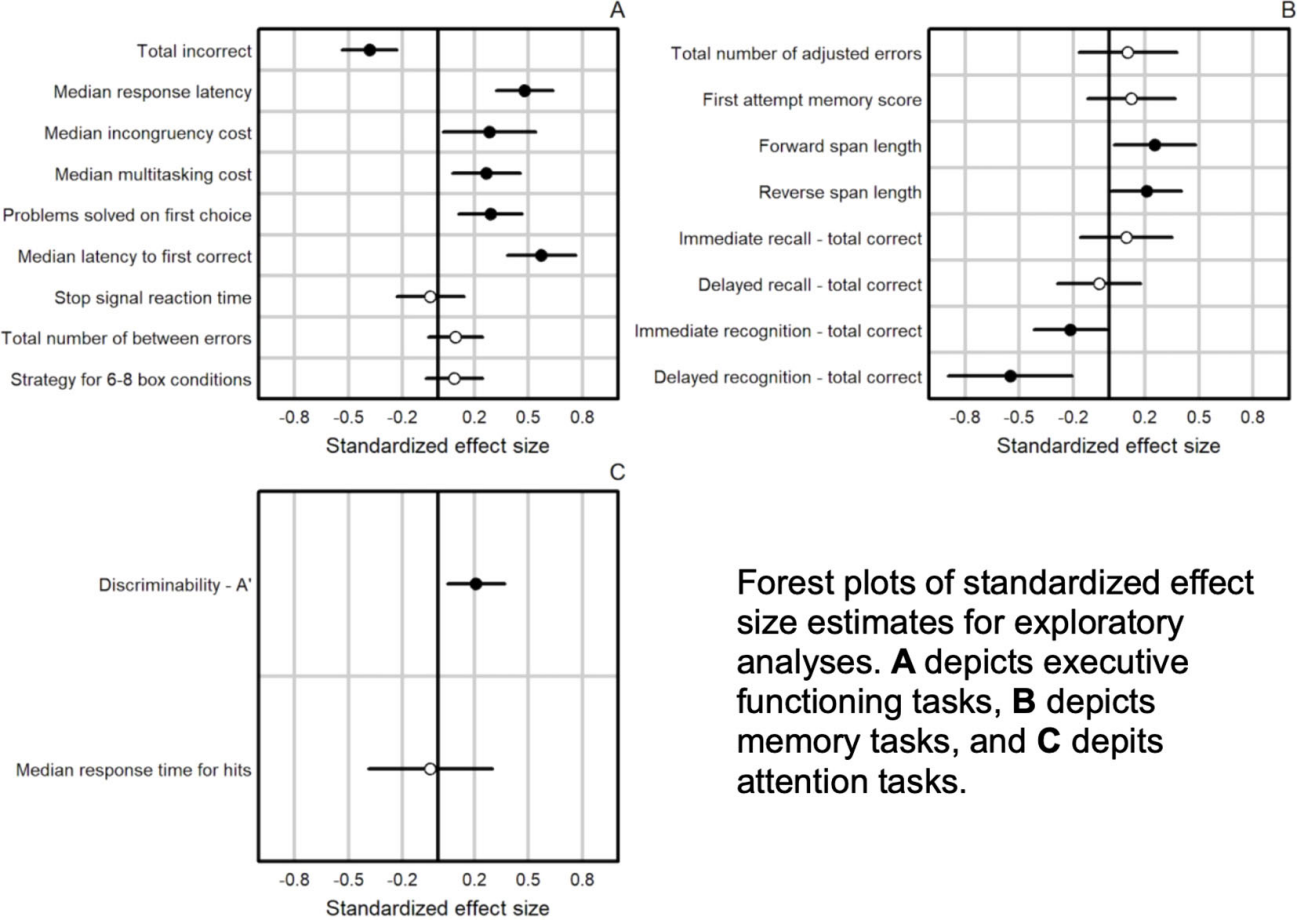
Summary of Exploratory Analyses.

Exploratory analyses assessed whether there were differences across all outcomes for post-baseline time points among participants who used vs those who did not use cannabis. The planned contrast indicated that CB-Abst performed differently from CB-Mon on stop signal reaction time post-baseline (p = 0.038; see **Figure 3**, **Supplementary Tables 4.1-4.3**). We did not find any significant differences between CB-Abst and NU (all p’s > 0.137) nor CB-Mon and NU (all p’s > 0.057) on any task post-baseline, implying that baseline differences between CB and NU resolved across the course of the study.

## Discussion

This study offers modest evidence that cannabis abstinence improves inhibitory control. At baseline, participants who used cannabis performed worse than their non-cannabis using peers on verbal recall and processing speed tasks. Cannabis abstinence was associated with improved inhibitory control over continued cannabis use. Adolescents who abstained from cannabis use for 4 weeks performed similarly to participants who did not use cannabis across most cognitive domains tested. Improvements in some tasks of x across all groups, suggests likely practice effects.

This study suggests that inhibitory control, an aspect of executive functioning involving suppression of an automatic or prepotent response, may be sensitive to early recovery after cannabis abstinence. Prior work implicates cannabis exposure with worsened inhibitory control (49) and broader executive functioning deficits (50). Young adults who use cannabis show poorer cross-sectional inhibitory control on Go/No-Go tasks and more impulsive decision-making during gambling tasks (51), as well as altered neural activation and connectivity in frontal, parietal, and cerebellar regions during go/no-go inhibitory control tasks (52–55). THC also impairs inhibitory control in acute administration studies by increasing premature responses and commission errors on reaction-time, maze, Go/No-Go, and Stop Signal tasks (56–60). This is the first study to our knowledge to demonstrate improved executive functioning and inhibitory control during early cannabis abstinence in adolescents.

Reduced inhibitory control in adolescence may lead to risky decision-making, reduced driving safety, and may reduce an individual’s ability to regulate their own drug use. The dual-process model of addiction posits that sensitization of reward circuitry, coupled with diminished top-down control, may underpin continued drug use (61).

In exploratory analyses, participants who abstained from cannabis performed similarly to those with no cannabis use by week 4 of the study, while participants who continued their usual cannabis use performed significantly worse on tasks measuring verbal recall and processing speed, suggesting the potential for a return to cognitive baseline with cannabis abstinence among adolescents who use cannabis regularly. Other research has suggested cannabis abstinence may be associated with cognitive, structural, and neurochemical recovery (62). Future research is needed to investigate whether there are sensitive periods or turning points during adolescence that facilitate or hinder cognitive recovery with cannabis initiation and abstinence.

We also observe that all groups in the study showed improvements on several of the assessed tasks, likely due to practice effects. In this study, practice effects were evenly distributed across groups and thus do not interfere with interpretation. However, this is an important methodological point for future research aiming to identify *acute* changes in cognition through repeated measures over time. Investigating questions about the specific timing of abstinence effects and cognitive improvement may be challenging as a result.

This study did not find hypothesized improvements in verbal learning with cannabis abstinence as observed among older adolescents and young adults (29). This study was conducted in younger participants with a different test battery. These design differences limit direct comparison across studies. In particular, the CANTAB was designed for an older population and may lack sensitivity to detect subtle changes in cognitive functioning within this younger age group, a limitation that has been pointed out in other studies (63).

## Limitations

Several limitations are worth considering. First, as noted above, the neurocognitive assessments used were not developed and age-normed for adolescents under age x which could have hampered sensitivity to detect performance changes with cannabis abstinence in this sample. Second, the 4-week window of cannabis abstinence evaluated here may be too brief to capture substantial changes in cognitive function. Additionally, NU participants were generally younger than the CB participants, reported less frequent alcohol use, and scored lower on the AUDIT at baseline. Although this might have been expected and may reflect patterns of age and symptoms among adolescents who do and do not use cannabis, it is possible that these differences contributed to variations in our cognitive variables of interest. Developmental differences between groups could have introduced residual confounds in between-group comparisons. We are also unable to account for other potential counfounds between cognitive functioning and cannabis use, for instance, education, grade level, GPA, or school engagement, which could presumably contribute to differences in cognitive functioning across groups and/or the impact of cannabis use on cognitive functioning. Additionally, although CB-Abst participants performed similarly to NU participants by Week 4, this improvement might reflect minimal change from baseline rather than true recovery. Baseline group differences and small within-group effect sizes suggest that this apparent “normalization” should be interpreted cautiously. Of note, the current study evaluates cannabis use along a binary (use versus no use), and does not account for the degree of use, which is becoming increasingly relevant as routes and doses of cannabis continue to change over time with development of new products and legalization. Differences in cannabis use frequency, route of administration, THC potency, and quantity of cannabis used during use sessions could influence changes in cognitive functioning, and future work would do well to consider these variables.

## Conclusion

This longitudinal experimental study demonstrated modest improvements in inhibitory control among adolescents who abstained from regular cannabis use for one month, compared to those who did not abstain. Further research is needed to prospectively assess cognition before and after cannabis initiation as well as to evaluate the impact of cannabis abstinence in a broader age range and over a longer duration of cannabis abstinence and to understand developmental impacts in the relationship between cannabis use, discontinuation, and cognitive functioning. With increasing adolescent cannabis use amid commercialization with proliferation of product types and increasing THC potency, it is essential to deepen our understanding of the effect of cannabis exposure and abstinence in adolescence on neurocognitive performance in order to pinpoint sensitive periods when intervention might produce the greatest cognitive benefits, supporting targeted prevention and recovery-focused care for adolescents who use cannabis.

## Data Availability

The raw data supporting the conclusions of this article will be made available by the authors, without undue reservation.
