# Supplementary material for: Neurocognitive outcomes in adolescents with and without four weeks of cannabis abstinence: a randomized clinical trial using contingency management
Source: Front Psychiatry. 2025 Dec 26;16:1723633. doi: 10.3389/fpsyt.2025.1723633 (PMC12785183; doi:10.3389/fpsyt.2025.1723633)
Supplement: Supplementary file 1 [file Table1.docx]

***Supplemental Table 1***

| **Modules Used to Assess Executive Functioning** | | | |
| --- | --- | --- | --- |
| Module | Task Description and Domain | Outcome | Model |
| Multitasking Test (MTT) | Participants had to indicate either the direction or position of an arrow for (a) blocks of trials in which the rule was the same or blocks of trials in which the rule varied from trial to trial, and (b) trials in which the direction and position of the arrow were either congruent or incongruent.  Domain: Tests ability to manage conflicting information and ignore task-irrelevant information. | - Total errors (total number of incorrect responses across all conditions)  - Ranges from 0 – 160  - Higher scores indicate worse performance | Binomial |
|  |  | - Median response latency (taken across all conditions)  - Ranges from 100 – 2000 milliseconds  - Higher scores indicate worse performance | Linear |
|  |  | - Median incongruency cost (Difference between incongruent and congruent trials)  - Ranges from -1900 – 1900 milliseconds  - Higher scores indicate worse performance | Linear |
|  |  | - Median multitasking cost (Difference between switching and non-switching blocks)  - Ranges from -1900 – 1900 milliseconds  - Higher scores indicate worse performance | Linear |
| One-Touch Stockings of Cambridge (OTS) | Participants had to indicate the number of moves it would take to rearrange a set of stacked balls.  Domain: Tests spatial planning and working memory. | - Total correct on first choice (indicated correct number of moves on first choice)  - Ranges from 0 – 15  - Higher scores indicate better performance | Binomial |
|  |  | - Median latency to correct choice (time it took to indicate correct number of moves on first choice)  - Ranges from 0 milliseconds and higher  - Higher scores indicate worse performance | Linear |
| Stop Signal Task (SST) | Participants had to indicate the direction of an arrow, but on a subset of trials they instead had to inhibit their response.  Domain: Tests response inhibition (impulse control). | - Stop signal reaction time (SSRT; the estimated time at which 50% of responses are inhibited)  - Ranges from 0 – 500 milliseconds  - Higher scores indicate worse performance | Linear |
| Spatial Working Memory (SWM) | Participants had to identify via process of elimination hidden tokens among a set of boxes.  Domain: Tests manipulation of visuospatial information, strategy, and working memory. | - Total number of between errors (times visited a box already known to have a token)  - Ranges from 0 – 63  - Higher scores indicate worse performance | Binomial |
|  |  | - Times used effective strategy (times person started from same box across trials)  - Ranges from 2 – 14  - Higher scores indicate better performance | Binomial |

| **Modules Used to Assess Memory** | | | |
| --- | --- | --- | --- |
| Module | Task description and Domain | Outcome | Model |
| Paired Associates Learning (PAL) | Participants had to indicate which box a token was hidden in, with multiple conditions with increasing number of boxes on screen.  Domain: Tests visual memory and new learning. | - Total number of errors (adjusted for number of uncompleted problems)  - Ranges from 0 – 70  - Higher scores indicate worse performance | Binomial |
|  |  | - First attempt memory score (number of times a person answered correctly on first try)  - Ranges from 0 – 20  - Higher scores indicate better performance | Binomial |
| Spatial Span (SSP) | Participants had to indicate the order (or the reverse order) in which a stimulus changed color.  Domain: Tests visuospatial working memory capacity. | - Forward span length (number of patterns person recalled in order successfully)  - Ranges from 2 – 9  - Higher scores indicate better performance | Binomial |
|  |  | - Reverse span length (number of patterns person recalled in reverse order successfully)  - Ranges from 2 – 9  - Higher scores indicate better performance | Binomial |
| Verbal Recognition Memory (VRM) | Participants studied a list of words and had to (a) recall the list, and (b) indicate which words were studied and which were new, following (1) a brief delay, and (2) a longer delay.  Domain: Tests verbal memory and new learning; ability to encode and subsequently retrieve verbal information. | - Total correct – immediate recall (words recalled immediately after studying)  - Ranges from 0 – 18  - Higher scores indicate better performance | Binomial |
|  |  | - Total correct – delayed recall (words recalled following delay after studying)  - Ranges from 0 – 18  - Higher scores indicate better performance | Binomial |
|  |  | - Total correct – immediate recognition (Words recognized immediately after studying)  - Ranges from 0 – 36  - Higher scores indicate better performance | Binomial |
|  |  | - Total correct – immediate recognition (Words recognized following delay after studying)  - Ranges from 0 – 36  - Higher scores indicate better performance | Binomial |
| **Module Used to Assess Attention** | | | |
| Module | Task description and Domain | Outcome | Model |
| Rapid Visual Information Processing (RVP) | While seeing a sequence of digits (100 per minute) participants had to indicate when they saw a target sequence of 3 digits.  Domain: Measures sustained attention. | - Discriminability (A’ computed from hits and false alarms)  - Ranges from 0 – 1  - Higher scores indicate better performance | Linear |
|  |  | - Median response time for hits (time to response when correctly identifying a target sequence)  - Ranges from 100 – 1900 milliseconds  - Higher scores indicate worse performance | Linear |

***Supplemental Table 2.1***

Planned Group Comparisons on Baseline Demographic Characteristics

| Domain | Measure | Contrast | Result |
| --- | --- | --- | --- |
| Samp char. | Reached intervention end; % (n) | CA ≠ CM | β = 0.52 [SE = 0.54]; p = 0.335 |
|  |  | CA ≠ NU | β = 0.94 [SE = 0.59]; p = 0.112 |
|  |  | CM ≠ NU | β = 0.42 [SE = 0.61]; p = 0.495 |
|  | Age; M (SD) | CA ≠ CM | β = -0.11 [SE = 0.29]; p = 0.695 |
|  |  | CA ≠ NU | β = -1.27 [SE = 0.29]; p < 0.001 |
|  |  | CM ≠ NU | β = -1.16 [SE = 0.27]; p < 0.001 |
|  | - Female | CA ≠ CM | β = -0.05 [SE = 0.37]; p = 0.898 |
|  |  | CA ≠ NU | β = 0.4 [SE = 0.37]; p = 0.279 |
|  |  | CM ≠ NU | β = 0.44 [SE = 0.35]; p = 0.202 |
|  | - Male | CA ≠ CM | β = 0.05 [SE = 0.37]; p = 0.898 |
|  |  | CA ≠ NU | β = -0.4 [SE = 0.37]; p = 0.279 |
|  |  | CM ≠ NU | β = -0.44 [SE = 0.35]; p = 0.202 |
|  | - American Indian/Alaska Native | CA ≠ CM | Cell counts too low |
|  |  | CA ≠ NU | Cell counts too low |
|  |  | CM ≠ NU | Cell counts too low |
|  | - Asian | CA ≠ CM | β = 0.39 [SE = 0.66]; p = 0.548 |
|  |  | CA ≠ NU | β = 0.17 [SE = 0.67]; p = 0.795 |
|  |  | CM ≠ NU | β = -0.22 [SE = 0.59]; p = 0.707 |
|  | - Black/African American | CA ≠ CM | β = -0.24 [SE = 0.49]; p = 0.623 |
|  |  | CA ≠ NU | β = 0.11 [SE = 0.46]; p = 0.806 |
|  |  | CM ≠ NU | β = 0.35 [SE = 0.46]; p = 0.436 |
|  | - More than one race | CA ≠ CM | β = 0.47 [SE = 0.54]; p = 0.388 |
|  |  | CA ≠ NU | β = -0.71 [SE = 0.67]; p = 0.292 |
|  |  | CM ≠ NU | β = -1.18 [SE = 0.61]; p = 0.054 |
|  | - Not listed | CA ≠ CM | β = 0.21 [SE = 0.93]; p = 0.819 |
|  |  | CA ≠ NU | β = 0.91 [SE = 0.84]; p = 0.279 |
|  |  | CM ≠ NU | β = 0.69 [SE = 0.73]; p = 0.342 |
|  | - White | CA ≠ CM | β = -0.24 [SE = 0.37]; p = 0.52 |
|  |  | CA ≠ NU | β = -0.04 [SE = 0.37]; p = 0.922 |
|  |  | CM ≠ NU | β = 0.2 [SE = 0.35]; p = 0.56 |
|  | Hispanic/Latino(a); % (n) | CA ≠ CM | β = 1.14 [SE = 0.55]; p = 0.038 |
|  |  | CA ≠ NU | β = 0.39 [SE = 0.59]; p = 0.514 |
|  |  | CM ≠ NU | β = -0.76 [SE = 0.46]; p = 0.098 |
|  | - Lifetime APSS; M (SD) | CA ≠ CM | β = 0.2 [SE = 0.12]; p = 0.093 |
|  |  | CA ≠ NU | β = 0.02 [SE = 0.12]; p = 0.898 |
|  |  | CM ≠ NU | β = -0.19 [SE = 0.11]; p = 0.098 |
|  | - MASQ; M (SD) | CA ≠ CM | β = 0.01 [SE = 0.03]; p = 0.658 |
|  |  | CA ≠ NU | β = -0.24 [SE = 0.03]; p < 0.001 |
|  |  | CM ≠ NU | β = -0.25 [SE = 0.03]; p < 0.001 |
|  | - SCARED GAD; M (SD) | CA ≠ CM | β = -0.01 [SE = 0.09]; p = 0.949 |
|  |  | CA ≠ NU | β = 0.1 [SE = 0.09]; p = 0.275 |
|  |  | CM ≠ NU | β = 0.1 [SE = 0.09]; p = 0.222 |

| Domain | Measure | Contrast | Result |
| --- | --- | --- | --- |
|  | - Years w/ cannabis use; M (SD) | CA ≠ CM | β = 0.1 [SE = 0.26]; p = 0.701 |
|  |  | CA ≠ NU | N/A |
|  |  | CM ≠ NU | N/A |
|  | - Days w/ cannabis use; M (SD) | CA ≠ CM | β = -0.1 [SE = 0.11]; p = 0.336 |
|  |  | CA ≠ NU | N/A |
|  |  | CM ≠ NU | N/A |
|  | - THCCOOH levels; M (SD) | CA ≠ CM | β = 177.8 [SE = 111.32]; p = 0.11 |
|  |  | CA ≠ NU | N/A |
|  |  | CM ≠ NU | N/A |
|  | - CUDIT-R; M (SD) | CA ≠ CM | β = 0.06 [SE = 0.07]; p = 0.377 |
|  |  | CA ≠ NU | N/A |
|  |  | CM ≠ NU | N/A |
|  | - Days w/ alcohol use; M (SD) | CA ≠ CM | β = -0.61 [SE = 0.26]; p = 0.018 |
|  |  | CA ≠ NU | β = -2.56 [SE = 0.53]; p < 0.001 |
|  |  | CM ≠ NU | β = -1.96 [SE = 0.54]; p < 0.001 |
|  | - AUDIT; M (SD) | CA ≠ CM | β = 0.11 [SE = 0.08]; p = 0.183 |
|  |  | CA ≠ NU | β = -1.11 [SE = 0.11]; p < 0.001 |
|  |  | CM ≠ NU | β = -1.22 [SE = 0.1]; p < 0.001 |

***Supplemental Table 2.2***

Planned Group Comparisons on Cognitive Performance Tasks

| Domain | Measure | Contrast | Result |
| --- | --- | --- | --- |
| Memory [PAL] | Total number of adjusted errors | CA vs. CM | β = -0.06 [SE = 0.07]; p = 0.369; ES = -0.09 [-0.51 to 0.31] |
| Memory [PAL] | Total number of adjusted errors | CA vs. NU | β = 0.07 [SE = 0.07]; p = 0.36; ES = -0.07 [-0.46 to 0.32] |
| Memory [PAL] | Total number of adjusted errors | CM vs. NU | β = 0.07 [SE = 0.07]; p = 0.36; ES = -0.08 [-0.5 to 0.33] |
| Memory [PAL] | First attempt memory score | CA vs. CM | β = 0.03 [SE = 0.07]; p = 0.691; ES = 0.01 [-0.47 to 0.46] |
| Memory [PAL] | First attempt memory score | CA vs. NU | β = -0.06 [SE = 0.07]; p = 0.443; ES = 0 [-0.46 to 0.43] |
| Memory [PAL] | First attempt memory score | CM vs. NU | β = -0.06 [SE = 0.08]; p = 0.443; ES = 0 [-0.45 to 0.46] |
| Memory [SSP] | Forward span length | CA vs. CM | β = 0.08 [SE = 0.07]; p = 0.241; ES = 0.17 [-0.2 to 0.53] |
| Memory [SSP] | Forward span length | CA vs. NU | β = -0.03 [SE = 0.07]; p = 0.696; ES = 0.18 [-0.24 to 0.56] |
| Memory [SSP] | Forward span length | CM vs. NU | β = -0.03 [SE = 0.07]; p = 0.696; ES = 0.18 [-0.2 to 0.59] |
| Memory [SSP] | Reverse span length | CA vs. CM | β = -0.01 [SE = 0.07]; p = 0.842; ES = -0.08 [-0.46 to 0.29] |
| Memory [SSP] | Reverse span length | CA vs. NU | β = -0.04 [SE = 0.07]; p = 0.585; ES = -0.08 [-0.46 to 0.29] |
| Memory [SSP] | Reverse span length | CM vs. NU | β = -0.04 [SE = 0.07]; p = 0.585; ES = -0.08 [-0.46 to 0.31] |
| Memory [VRM] | Immediate recall - total correct | CA vs. CM | β = 0 [SE = 0.04]; p = 0.944; ES = 0.29 [-0.15 to 0.74] |
| Memory [VRM] | Immediate recall - total correct | CA vs. NU | β = 0.13 [SE = 0.04]; p = 0.001; ES = 0.31 [-0.12 to 0.73] |
| Memory [VRM] | Immediate recall - total correct | CM vs. NU | β = 0.14 [SE = 0.04]; p = 0.001; ES = 0.29 [-0.12 to 0.7] |
| Memory [VRM] | Delayed recall - total correct | CA vs. CM | β = -0.01 [SE = 0.04]; p = 0.807; ES = 0.18 [-0.18 to 0.54] |
| Memory [VRM] | Delayed recall - total correct | CA vs. NU | β = 0.11 [SE = 0.04]; p = 0.006; ES = 0.17 [-0.19 to 0.52] |
| Memory [VRM] | Delayed recall - total correct | CM vs. NU | β = 0.11 [SE = 0.04]; p = 0.006; ES = 0.19 [-0.16 to 0.55] |
| Memory [VRM] | Immediate recognition - total correct | CA vs. CM | β = 0.1 [SE = 0.07]; p = 0.162; ES = 0.13 [-0.31 to 0.55] |
| Memory [VRM] | Immediate recognition - total correct | CA vs. NU | β = -0.1 [SE = 0.07]; p = 0.142; ES = 0.13 [-0.27 to 0.56] |
| Memory [VRM] | Immediate recognition - total correct | CM vs. NU | β = -0.1 [SE = 0.07]; p = 0.142; ES = 0.12 [-0.31 to 0.53] |
| Memory [VRM] | Delayed recognition - total correct | CA vs. CM | β = 0.07 [SE = 0.07]; p = 0.309; ES = 0.11 [-0.32 to 0.54] |
| Memory [VRM] | Delayed recognition - total correct | CA vs. NU | β = -0.06 [SE = 0.06]; p = 0.336; ES = 0.1 [-0.31 to 0.51] |
| Memory [VRM] | Delayed recognition - total correct | CM vs. NU | β = -0.07 [SE = 0.07]; p = 0.336; ES = 0.09 [-0.36 to 0.55] |
| Executive functioning [MTT] | Total incorrect | CA vs. CM | β = 0.01 [SE = 0.07]; p = 0.934; ES = 0.06 [-0.27 to 0.4] |
| Executive functioning [MTT] | Total incorrect | CA vs. NU | β = 0.05 [SE = 0.07]; p = 0.455; ES = 0.06 [-0.26 to 0.38] |
| Executive functioning [MTT] | Total incorrect | CM vs. NU | β = 0.05 [SE = 0.07]; p = 0.455; ES = 0.06 [-0.24 to 0.38] |
| Executive functioning [MTT] | Median response latency (ms) | CA vs. CM | β = -12.6 [SE = 7.1]; p = 0.075; ES = -0.12 [-0.56 to 0.35] |
| Executive functioning [MTT] | Median response latency (ms) | CA vs. NU | β = 17.7 [SE = 7.1]; p = 0.012; ES = -0.12 [-0.54 to 0.31] |
| Executive functioning [MTT] | Median response latency (ms) | CM vs. NU | β = 18.6 [SE = 7.4]; p = 0.012; ES = -0.11 [-0.56 to 0.33] |
| Executive functioning [MTT] | Median incongruency cost (ms) | CA vs. CM | β = 1.3 [SE = 3.1]; p = 0.682; ES = 0.26 [-0.21 to 0.7] |
| Executive functioning [MTT] | Median incongruency cost (ms) | CA vs. NU | β = 5.3 [SE = 3.1]; p = 0.084; ES = 0.26 [-0.17 to 0.73] |
| Executive functioning [MTT] | Median incongruency cost (ms) | CM vs. NU | β = 5.6 [SE = 3.2]; p = 0.084; ES = 0.24 [-0.21 to 0.67] |
| Executive functioning [MTT] | Median multitasking cost (ms) | CA vs. CM | β = -14.3 [SE = 7.6]; p = 0.06; ES = -0.27 [-0.75 to 0.18] |
| Executive functioning [MTT] | Median multitasking cost (ms) | CA vs. NU | β = 9 [SE = 7.6]; p = 0.232; ES = -0.26 [-0.71 to 0.22] |
| Executive functioning [MTT] | Median multitasking cost (ms) | CM vs. NU | β = 9.5 [SE = 8]; p = 0.232; ES = -0.26 [-0.72 to 0.21] |
| Executive functioning [OTS] | Problems solved on first choice | CA vs. CM | β = -0.09 [SE = 0.05]; p = 0.071; ES = -0.34 [-0.67 to -0.03] |
| Executive functioning [OTS] | Problems solved on first choice | CA vs. NU | β = -0.06 [SE = 0.05]; p = 0.247; ES = -0.34 [-0.65 to -0.01] |
| Executive functioning [OTS] | Problems solved on first choice | CM vs. NU | β = -0.06 [SE = 0.05]; p = 0.247; ES = -0.35 [-0.68 to -0.03] |
| Executive functioning [OTS] | Median latency to first correct choice (ms) | CA vs. CM | β = -284.7 [SE = 266.6]; p = 0.286; ES = -0.18 [-0.62 to 0.32] |
| Executive functioning [OTS] | Median latency to first correct choice (ms) | CA vs. NU | β = 131.3 [SE = 266.6]; p = 0.622; ES = -0.17 [-0.62 to 0.31] |
| Executive functioning [OTS] | Median latency to first correct choice (ms) | CM vs. NU | β = 141.3 [SE = 280.6]; p = 0.615; ES = -0.17 [-0.62 to 0.3] |
| Executive functioning [SST] | Stop signal reaction time (ms) | CA vs. CM | β = -4 [SE = 4.2]; p = 0.342; ES = -0.07 [-0.5 to 0.39] |
| Executive functioning [SST] | Stop signal reaction time (ms) | CA vs. NU | β = 5.2 [SE = 4.2]; p = 0.215; ES = -0.07 [-0.55 to 0.38] |
| Executive functioning [SST] | Stop signal reaction time (ms) | CM vs. NU | β = 5.5 [SE = 4.5]; p = 0.215; ES = -0.08 [-0.55 to 0.4] |
| Executive functioning [SWM] | Total number of between errors | CA vs. CM | β = -0.09 [SE = 0.1]; p = 0.39; ES = 0.09 [-0.34 to 0.52] |
| Executive functioning [SWM] | Total number of between errors | CA vs. NU | β = 0.28 [SE = 0.1]; p = 0.004; ES = 0.08 [-0.34 to 0.49] |
| Executive functioning [SWM] | Total number of between errors | CM vs. NU | β = 0.29 [SE = 0.1]; p = 0.004; ES = 0.08 [-0.35 to 0.49] |
| Executive functioning [SWM] | Strategy for 6-8 box conditions | CA vs. CM | β = -0.1 [SE = 0.05]; p = 0.049; ES = -0.09 [-0.4 to 0.21] |
| Executive functioning [SWM] | Strategy for 6-8 box conditions | CA vs. NU | β = 0.13 [SE = 0.05]; p = 0.007; ES = -0.08 [-0.38 to 0.19] |
| Executive functioning [SWM] | Strategy for 6-8 box conditions | CM vs. NU | β = 0.14 [SE = 0.05]; p = 0.007; ES = -0.09 [-0.39 to 0.22] |
| Attention [RVP] | Discriminability - A' | CA vs. CM | β = 0.002 [SE = 0.005]; p = 0.71; ES = -0.18 [-0.64 to 0.29] |
| Attention [RVP] | Discriminability - A' | CA vs. NU | β = -0.012 [SE = 0.005]; p = 0.009; ES = -0.19 [-0.6 to 0.23] |
| Attention [RVP] | Discriminability - A' | CM vs. NU | β = -0.013 [SE = 0.005]; p = 0.009; ES = -0.18 [-0.64 to 0.27] |
| Attention [RVP] | Median response time for hits (ms) | CA vs. CM | β = -4.4 [SE = 4.7]; p = 0.347; ES = -0.18 [-0.61 to 0.28] |
| Attention [RVP] | Median response time for hits (ms) | CA vs. NU | β = 0.7 [SE = 4.7]; p = 0.879; ES = -0.17 [-0.63 to 0.29] |
| Attention [RVP] | Median response time for hits (ms) | CM vs. NU | β = 0.7 [SE = 4.9]; p = 0.879; ES = -0.16 [-0.61 to 0.33] |

***Supplemental Table 3***

Group x Time Interaction and Main Effect of Time

| Domain | Outcome | Type | Test |
| --- | --- | --- | --- |
| Memory [PAL] | Total number of adjusted errors | Group x Time interaction | χ²(1) = 0.07; p = 0.798 |
| Memory [PAL] | Total number of adjusted errors | Main effect of time | β = -0.62 [SE = 0.73]; p = 0.393 (α = 0.025); ES = -0.02 [-0.09 to 0.05] |
| Memory [PAL] | First attempt memory score | Group x Time interaction | χ²(1) = 0.11; p = 0.739 |
| Memory [PAL] | First attempt memory score | Main effect of time | β = 0.37 [SE = 0.31]; p = 0.246 (α = 0.025); ES = 0 [0 to 0] |
| Memory [SSP] | Forward span length | Group x Time interaction | χ²(1) = 2.09; p = 0.149 |
| Memory [SSP] | Forward span length | Main effect of time | β = 0.35 [SE = 0.14]; p = 0.013 (α = 0.025); ES = 0.01 [0 to 0.01] |
| Memory [SSP] | Reverse span length | Group x Time interaction | χ²(1) = 0.1; p = 0.751 |
| Memory [SSP] | Reverse span length | Main effect of time | β = 0.3 [SE = 0.12]; p = 0.014 (α = 0.025); ES = 0.01 [0 to 0.02] |
| Memory [VRM] | Immediate recall - total correct | Group x Time interaction | χ²(1) = 0.1; p = 0.757 |
| Memory [VRM] | Immediate recall - total correct | Main effect of time | β = 0.2 [SE = 0.21]; p = 0.354 (α = 0.0125); ES = 0 [0 to 0] |
| Memory [VRM] | Delayed recall - total correct | Group x Time interaction | χ²(1) = 0.14; p = 0.709 |
| Memory [VRM] | Delayed recall - total correct | Main effect of time | β = -0.15 [SE = 0.24]; p = 0.541 (α = 0.0125); ES = 0 [-0.01 to 0] |
| Memory [VRM] | Immediate recognition - total correct | Group x Time interaction | χ²(1) = 0.11; p = 0.740 |
| Memory [VRM] | Immediate recognition - total correct | Main effect of time | β = -0.64 [SE = 0.25]; p = 0.009 (α = 0.0125); ES = 0 [0 to 0] |
| Memory [VRM] | Delayed recognition - total correct | Group x Time interaction | χ²(1) = 0.06; p = 0.801 |
| Memory [VRM] | Delayed recognition - total correct | Main effect of time | β = -1.43 [SE = 0.34]; p < 0.001 (α = 0.0125); ES = 0 [0 to 0] |
| Executive functioning [MTT] | Total incorrect | Group x Time interaction | χ²(1) = 0.12; p = 0.732 |
| Executive functioning [MTT] | Total incorrect | Main effect of time | β = 2.8 [SE = 0.47]; p < 0.001 (α = 0.0125); ES = 0.03 [0 to 0.07] |
| Executive functioning [MTT] | Median response latency (ms) | Group x Time interaction | χ²(1) = 0.08; p = 0.780 |
| Executive functioning [MTT] | Median response latency (ms) | Main effect of time | β = -42.3 [SE = 5.4]; p < 0.001 (α = 0.0125); ES = -0.48 [-0.63 to -0.33] |
| Executive functioning [MTT] | Median incongruency cost (ms) | Group x Time interaction | χ²(1) = 0.5; p = 0.481 |
| Executive functioning [MTT] | Median incongruency cost (ms) | Main effect of time | β = -9.3 [SE = 3.3]; p = 0.006 (α = 0.0125); ES = -0.28 [-0.54 to -0.03] |
| Executive functioning [MTT] | Median multitasking cost (ms) | Group x Time interaction | χ²(1) = 0.41; p = 0.521 |
| Executive functioning [MTT] | Median multitasking cost (ms) | Main effect of time | β = -24 [SE = 6.5]; p < 0.001 (α = 0.0125); ES = -0.27 [-0.45 to -0.09] |
| Executive functioning [OTS] | Problems solved on first choice | Group x Time interaction | χ²(1) = 0.7; p = 0.401 |
| Executive functioning [OTS] | Problems solved on first choice | Main effect of time | β = 0.6 [SE = 0.16]; p < 0.001 (α = 0.025); ES = 0 [0 to 0] |
| Executive functioning [OTS] | Median latency to first correct choice (ms) | Group x Time interaction | χ²(1) = 3.46; p = 0.063 |
| Executive functioning [OTS] | Median latency to first correct choice (ms) | Main effect of time | β = -1835.3 [SE = 271.4]; p < 0.001 (α = 0.025); ES = -0.57 [-0.76 to -0.38] |
| Executive functioning [SST] | Stop signal reaction time (ms) | Group x Time interaction | χ²(1) = 1.47; p = 0.226 |
| Executive functioning [SST] | Stop signal reaction time (ms) | Main effect of time | β = 2 [SE = 4.4]; p = 0.643 (α = 0.05); ES = 0.04 [-0.14 to 0.23] |
| Executive functioning [SWM] | Total number of between errors | Group x Time interaction | χ²(1) = 0.25; p = 0.615 |
| Executive functioning [SWM] | Total number of between errors | Main effect of time | β = -0.79 [SE = 0.53]; p = 0.140 (α = 0.025); ES = -0.12 [-0.35 to 0.11] |
| Executive functioning [SWM] | Strategy for 6-8 box conditions | Group x Time interaction | χ²(1) = 0.81; p = 0.368 |
| Executive functioning [SWM] | Strategy for 6-8 box conditions | Main effect of time | β = -0.23 [SE = 0.18]; p = 0.212 (α = 0.025); ES = -0.03 [-0.08 to 0.02] |
| Attention [RVP] | Discriminability - A' | Group x Time interaction | χ²(1) = 0.38; p = 0.539 |
| Attention [RVP] | Discriminability - A' | Main effect of time | β = 0.011 [SE = 0.004]; p = 0.003 (α = 0.025); ES = 0.21 [0.05 to 0.36] |
| Attention [RVP] | Median response time for hits (ms) | Group x Time interaction | χ²(1) = 1.51; p = 0.219 |
| Attention [RVP] | Median response time for hits (ms) | Main effect of time | β = 2.7 [SE = 9.2]; p = 0.771 (α = 0.025); ES = 0.04 [-0.3 to 0.39] |

***Supplemental Table 4.1***

Results for main effect of group and pairwise comparisons for CB-Abst, CB-Mon, and No Use groups

| Domain | Outcome | Type | Test |
| --- | --- | --- | --- |
| Memory [PAL] | Total number of adjusted errors | Main effect of group | χ²(2) = 1.19; p = 0.304 |
| Memory [PAL] | Total number of adjusted errors | CB-Abst vs. CB-Mon | β = -0.21 [SE = 0.16]; p = 0.198 (α = 0.025); ES = -0.18 [-0.49 to 0.13] |
| Memory [PAL] | Total number of adjusted errors | CB-Abst vs. Non-User | β = -0.01 [SE = 0.18]; p = 0.977 (α = 0.025); ES = 0 [-0.31 to 0.3] |
| Memory [PAL] | Total number of adjusted errors | CB-Mon vs. Non-User | β = 0.21 [SE = 0.14]; p = 0.135 (α = 0.025); ES = 0.18 [-0.09 to 0.44] |
| Memory [PAL] | First attempt memory score | Main effect of group | χ²(2) = 0.67; p = 0.511 |
| Memory [PAL] | First attempt memory score | CB-Abst vs. CB-Mon | β = 0.09 [SE = 0.14]; p = 0.505 (α = 0.025); ES = 0.09 [-0.21 to 0.4] |
| Memory [PAL] | First attempt memory score | CB-Abst vs. Non-User | β = -0.07 [SE = 0.15]; p = 0.641 (α = 0.025); ES = -0.06 [-0.37 to 0.25] |
| Memory [PAL] | First attempt memory score | CB-Mon vs. Non-User | β = -0.16 [SE = 0.13]; p = 0.208 (α = 0.025); ES = -0.16 [-0.43 to 0.12] |
| Memory [SSP] | Forward span length | Main effect of group | χ²(2) = 2.6; p = 0.074 |
| Memory [SSP] | Forward span length | CB-Abst vs. CB-Mon | β = 0.3 [SE = 0.14]; p = 0.037 (α = 0.025); ES = 0.25 [-0.01 to 0.51] |
| Memory [SSP] | Forward span length | CB-Abst vs. Non-User | β = 0.05 [SE = 0.16]; p = 0.772 (α = 0.025); ES = 0.04 [-0.24 to 0.32] |
| Memory [SSP] | Forward span length | CB-Mon vs. Non-User | β = -0.25 [SE = 0.13]; p = 0.057 (α = 0.025); ES = -0.21 [-0.46 to 0.03] |
| Memory [SSP] | Reverse span length | Main effect of group | χ²(2) = 0.24; p = 0.787 |
| Memory [SSP] | Reverse span length | CB-Abst vs. CB-Mon | β = -0.08 [SE = 0.13]; p = 0.529 (α = 0.025); ES = -0.08 [-0.37 to 0.21] |
| Memory [SSP] | Reverse span length | CB-Abst vs. Non-User | β = -0.05 [SE = 0.12]; p = 0.649 (α = 0.025); ES = -0.05 [-0.32 to 0.21] |
| Memory [SSP] | Reverse span length | CB-Mon vs. Non-User | β = 0.03 [SE = 0.11]; p = 0.799 (α = 0.025); ES = 0.03 [-0.22 to 0.27] |
| Memory [VRM] | Immediate recall - total correct | Main effect of group | χ²(2) = 0.69; p = 0.502 |
| Memory [VRM] | Immediate recall - total correct | CB-Abst vs. CB-Mon | β = 0.09 [SE = 0.08]; p = 0.251 (α = 0.0125); ES = 0.17 [-0.2 to 0.55] |
| Memory [VRM] | Immediate recall - total correct | CB-Abst vs. Non-User | β = 0.07 [SE = 0.08]; p = 0.371 (α = 0.0125); ES = 0.14 [-0.26 to 0.55] |
| Memory [VRM] | Immediate recall - total correct | CB-Mon vs. Non-User | β = -0.01 [SE = 0.07]; p = 0.842 (α = 0.0125); ES = -0.03 [-0.39 to 0.34] |
| Memory [VRM] | Delayed recall - total correct | Main effect of group | χ²(2) = 0.58; p = 0.562 |
| Memory [VRM] | Delayed recall - total correct | CB-Abst vs. CB-Mon | β = 0.08 [SE = 0.08]; p = 0.359 (α = 0.0125); ES = 0.13 [-0.22 to 0.47] |
| Memory [VRM] | Delayed recall - total correct | CB-Abst vs. Non-User | β = 0 [SE = 0.09]; p = 0.969 (α = 0.0125); ES = -0.01 [-0.39 to 0.37] |
| Memory [VRM] | Delayed recall - total correct | CB-Mon vs. Non-User | β = -0.08 [SE = 0.09]; p = 0.365 (α = 0.0125); ES = -0.13 [-0.49 to 0.23] |
| Memory [VRM] | Immediate recognition - total correct | Main effect of group | χ²(2) = 0.79; p = 0.454 |
| Memory [VRM] | Immediate recognition - total correct | CB-Abst vs. CB-Mon | β = 0.08 [SE = 0.12]; p = 0.472 (α = 0.0125); ES = 0.1 [-0.24 to 0.44] |
| Memory [VRM] | Immediate recognition - total correct | CB-Abst vs. Non-User | β = 0.15 [SE = 0.1]; p = 0.137 (α = 0.0125); ES = 0.18 [-0.12 to 0.48] |
| Memory [VRM] | Immediate recognition - total correct | CB-Mon vs. Non-User | β = 0.07 [SE = 0.11]; p = 0.561 (α = 0.0125); ES = 0.08 [-0.27 to 0.43] |
| Memory [VRM] | Delayed recognition - total correct | Main effect of group | χ²(2) = 0.31; p = 0.733 |
| Memory [VRM] | Delayed recognition - total correct | CB-Abst vs. CB-Mon | β = -0.06 [SE = 0.09]; p = 0.475 (α = 0.0125); ES = -0.11 [-0.49 to 0.27] |
| Memory [VRM] | Delayed recognition - total correct | CB-Abst vs. Non-User | β = 0.01 [SE = 0.1]; p = 0.928 (α = 0.0125); ES = 0.02 [-0.42 to 0.46] |
| Memory [VRM] | Delayed recognition - total correct | CB-Mon vs. Non-User | β = 0.07 [SE = 0.11]; p = 0.488 (α = 0.0125); ES = 0.12 [-0.33 to 0.57] |

***Supplemental Table 4.2***

Results for main effect of group and pairwise comparisons for CB-Abst, CB-Mon, and No Use groups

| Domain | Outcome | Type | Test |
| --- | --- | --- | --- |
| Executive functioning [MTT] | Total incorrect | Main effect of group | χ²(2) = 1.19; p = 0.305 |
| Executive functioning [MTT] | Total incorrect | CB-Abst vs. CB-Mon | β = -0.11 [SE = 0.13]; p = 0.413 (α = 0.0125); ES = -0.11 [-0.46 to 0.23] |
| Executive functioning [MTT] | Total incorrect | CB-Abst vs. Non-User | β = -0.23 [SE = 0.13]; p = 0.076 (α = 0.0125); ES = -0.26 [-0.61 to 0.1] |
| Executive functioning [MTT] | Total incorrect | CB-Mon vs. Non-User | β = -0.12 [SE = 0.11]; p = 0.266 (α = 0.0125); ES = -0.14 [-0.46 to 0.18] |
| Executive functioning [MTT] | Median response latency (ms) | Main effect of group | χ²(2) = 0.14; p = 0.866 |
| Executive functioning [MTT] | Median response latency (ms) | CB-Abst vs. CB-Mon | β = 2.1 [SE = 9.2]; p = 0.820 (α = 0.0125); ES = 0.02 [-0.23 to 0.28] |
| Executive functioning [MTT] | Median response latency (ms) | CB-Abst vs. Non-User | β = 5.4 [SE = 9.7]; p = 0.583 (α = 0.0125); ES = 0.06 [-0.21 to 0.34] |
| Executive functioning [MTT] | Median response latency (ms) | CB-Mon vs. Non-User | β = 3.3 [SE = 9.7]; p = 0.735 (α = 0.0125); ES = 0.04 [-0.24 to 0.31] |
| Executive functioning [MTT] | Median incongruency cost (ms) | Main effect of group | χ²(2) = 0.19; p = 0.826 |
| Executive functioning [MTT] | Median incongruency cost (ms) | CB-Abst vs. CB-Mon | β = -0.6 [SE = 4]; p = 0.888 (α = 0.0125); ES = -0.01 [-0.28 to 0.25] |
| Executive functioning [MTT] | Median incongruency cost (ms) | CB-Abst vs. Non-User | β = -2.4 [SE = 4]; p = 0.548 (α = 0.0125); ES = -0.06 [-0.33 to 0.2] |
| Executive functioning [MTT] | Median incongruency cost (ms) | CB-Mon vs. Non-User | β = -1.9 [SE = 3.5]; p = 0.601 (α = 0.0125); ES = -0.05 [-0.28 to 0.18] |
| Executive functioning [MTT] | Median multitasking cost (ms) | Main effect of group | χ²(2) = 1.74; p = 0.176 |
| Executive functioning [MTT] | Median multitasking cost (ms) | CB-Abst vs. CB-Mon | β = 16.7 [SE = 9.4]; p = 0.076 (α = 0.0125); ES = 0.18 [-0.07 to 0.43] |
| Executive functioning [MTT] | Median multitasking cost (ms) | CB-Abst vs. Non-User | β = 15.8 [SE = 9.7]; p = 0.102 (α = 0.0125); ES = 0.17 [-0.09 to 0.43] |
| Executive functioning [MTT] | Median multitasking cost (ms) | CB-Mon vs. Non-User | β = -0.8 [SE = 8.9]; p = 0.925 (α = 0.0125); ES = -0.01 [-0.24 to 0.23] |
| Executive functioning [OTS] | Problems solved on first choice | Main effect of group | χ²(2) = 1.25; p = 0.288 |
| Executive functioning [OTS] | Problems solved on first choice | CB-Abst vs. CB-Mon | β = 0.15 [SE = 0.1]; p = 0.150 (α = 0.025); ES = 0.16 [-0.09 to 0.41] |
| Executive functioning [OTS] | Problems solved on first choice | CB-Abst vs. Non-User | β = 0.14 [SE = 0.11]; p = 0.212 (α = 0.025); ES = 0.15 [-0.13 to 0.43] |
| Executive functioning [OTS] | Problems solved on first choice | CB-Mon vs. Non-User | β = -0.01 [SE = 0.12]; p = 0.958 (α = 0.025); ES = -0.01 [-0.31 to 0.3] |
| Executive functioning [OTS] | Median latency to first correct choice (ms) | Main effect of group | χ²(2) = 0.17; p = 0.846 |
| Executive functioning [OTS] | Median latency to first correct choice (ms) | CB-Abst vs. CB-Mon | β = 173.4 [SE = 301.3]; p = 0.565 (α = 0.025); ES = 0.05 [-0.15 to 0.26] |
| Executive functioning [OTS] | Median latency to first correct choice (ms) | CB-Abst vs. Non-User | β = 175.3 [SE = 324.2]; p = 0.589 (α = 0.025); ES = 0.05 [-0.17 to 0.27] |
| Executive functioning [OTS] | Median latency to first correct choice (ms) | CB-Mon vs. Non-User | β = 1.9 [SE = 309.5]; p = 0.995 (α = 0.025); ES = 0 [-0.21 to 0.21] |
| Executive functioning [SST] | Stop signal reaction time (ms) | Main effect of group | χ²(2) = 2.38; p = 0.093 |
| Executive functioning [SST] | Stop signal reaction time (ms) | CB-Abst vs. CB-Mon | β = -10.7 [SE = 5.1]; p = 0.038 (α = 0.05); ES = -0.2 [-0.4 to -0.01] |
| Executive functioning [SST] | Stop signal reaction time (ms) | CB-Abst vs. Non-User | β = -8.8 [SE = 5.5]; p = 0.108 (α = 0.05); ES = -0.17 [-0.37 to 0.04] |
| Executive functioning [SST] | Stop signal reaction time (ms) | CB-Mon vs. Non-User | β = 1.9 [SE = 6.6]; p = 0.775 (α = 0.05); ES = 0.04 [-0.21 to 0.28] |
| Executive functioning [SWM] | Total number of between errors | Main effect of group | χ²(2) = 0.43; p = 0.651 |
| Executive functioning [SWM] | Total number of between errors | CB-Abst vs. CB-Mon | β = -0.12 [SE = 0.16]; p = 0.452 (α = 0.025); ES = -0.06 [-0.23 to 0.11] |
| Executive functioning [SWM] | Total number of between errors | CB-Abst vs. Non-User | β = 0 [SE = 0.19]; p = 0.991 (α = 0.025); ES = 0 [-0.19 to 0.19] |
| Executive functioning [SWM] | Total number of between errors | CB-Mon vs. Non-User | β = 0.12 [SE = 0.18]; p = 0.486 (α = 0.025); ES = 0.06 [-0.13 to 0.25] |
| Executive functioning [SWM] | Strategy for 6-8 box conditions | Main effect of group | χ²(2) = 0.67; p = 0.510 |
| Executive functioning [SWM] | Strategy for 6-8 box conditions | CB-Abst vs. CB-Mon | β = -0.16 [SE = 0.15]; p = 0.286 (α = 0.025); ES = -0.15 [-0.47 to 0.17] |
| Executive functioning [SWM] | Strategy for 6-8 box conditions | CB-Abst vs. Non-User | β = -0.12 [SE = 0.14]; p = 0.410 (α = 0.025); ES = -0.11 [-0.41 to 0.19] |
| Executive functioning [SWM] | Strategy for 6-8 box conditions | CB-Mon vs. Non-User | β = 0.04 [SE = 0.13]; p = 0.744 (α = 0.025); ES = 0.04 [-0.24 to 0.32] |

***Supplemental Table 4.3***

Results for main effect of group and pairwise comparisons for CB-Abst, CB-Mon, and No Use groups

| Domain | Outcome | Type | Test |
| --- | --- | --- | --- |
| Attention [RVP] | Discriminability - A' | Main effect of group | χ²(2) = 0.36; p = 0.695 |
| Attention [RVP] | Discriminability - A' | CB-Abst vs. CB-Mon | β = 0.001 [SE = 0.006]; p = 0.816 (α = 0.025); ES = 0.02 [-0.21 to 0.25] |
| Attention [RVP] | Discriminability - A' | CB-Abst vs. Non-User | β = -0.004 [SE = 0.007]; p = 0.562 (α = 0.025); ES = -0.07 [-0.33 to 0.2] |
| Attention [RVP] | Discriminability - A' | CB-Mon vs. Non-User | β = -0.005 [SE = 0.006]; p = 0.388 (α = 0.025); ES = -0.09 [-0.33 to 0.15] |
| Attention [RVP] | Median response time for hits (ms) | Main effect of group | χ²(2) = 0.84; p = 0.430 |
| Attention [RVP] | Median response time for hits (ms) | CB-Abst vs. CB-Mon | β = -11.4 [SE = 9.1]; p = 0.212 (α = 0.025); ES = -0.2 [-0.55 to 0.16] |
| Attention [RVP] | Median response time for hits (ms) | CB-Abst vs. Non-User | β = -7.5 [SE = 7.5]; p = 0.316 (α = 0.025); ES = -0.13 [-0.42 to 0.16] |
| Attention [RVP] | Median response time for hits (ms) | CB-Mon vs. Non-User | β = 3.8 [SE = 10]; p = 0.702 (α = 0.025); ES = 0.07 [-0.32 to 0.46] |
